# Noninvasive detection of Zika virus in mosquito excreta sampled from wild mosquito populations in French Guiana

**DOI:** 10.1093/jme/tjae016

**Published:** 2024-02-26

**Authors:** Amandine Guidez, Albin Fontaine, Léna Yousfi, Sara Moutailler, Romuald Carinci, Jean Issaly, Pascal Gaborit, Arnaud Cannet, Franck de Laval, Séverine Matheus, Dominique Rousset, Isabelle Dusfour, Romain Girod, Sébastien Briolant

**Affiliations:** Unité d’Entomologie Médicale, Institut Pasteur de la Guyane, Cayenne, French Guiana; Unité Parasitologie et Entomologie, Département de Microbiologie et Maladies Infectieuses, Institut de Recherche Biomédicale des Armées (IRBA), 19-21 Boulevard Jean Moulin,13005 Marseille, France; Aix Marseille Univ, IRD, SSA, AP-HM, UMR Vecteurs – Infections Tropicales et Méditerranéennes (VITROME), Marseille, France; Institut Hospitalo-Universitaire (IHU)–Méditerranée Infection, Marseille, France; ANSES, INRAE, Ecole Nationale Vétérinaire d’Alfort, UMR BIPAR, Laboratoire de Santé Animale, Maisons-Alfort F-94700, France; ANSES, INRAE, Ecole Nationale Vétérinaire d’Alfort, UMR BIPAR, Laboratoire de Santé Animale, Maisons-Alfort F-94700, France; Unité d’Entomologie Médicale, Institut Pasteur de la Guyane, Cayenne, French Guiana; Unité d’Entomologie Médicale, Institut Pasteur de la Guyane, Cayenne, French Guiana; Unité d’Entomologie Médicale, Institut Pasteur de la Guyane, Cayenne, French Guiana; CNEV, IRD, 34000 Montpellier, France; French Army Center for Epidemiology and Public Health (CESPA), Marseille, France; Centre National de Référence des Arbovirus, laboratoire associé, Institut Pasteur de la Guyane, Cayenne, French Guiana; Centre National de Référence des Arbovirus, laboratoire associé, Institut Pasteur de la Guyane, Cayenne, French Guiana; MIVEGEC, UMR IRD 224-CNRS 5290, Université de Montpellier, Montpellier, France; Département de Santé Globale, Institut Pasteur, Paris, France; Unité d’Entomologie Médicale, Institut Pasteur de la Guyane, Cayenne, French Guiana; Unité Parasitologie et Entomologie, Département de Microbiologie et Maladies Infectieuses, Institut de Recherche Biomédicale des Armées (IRBA), 19-21 Boulevard Jean Moulin,13005 Marseille, France; Aix Marseille Univ, IRD, SSA, AP-HM, UMR Vecteurs – Infections Tropicales et Méditerranéennes (VITROME), Marseille, France; Institut Hospitalo-Universitaire (IHU)–Méditerranée Infection, Marseille, France

**Keywords:** arbovirus surveillance, Zika virus, detection, mosquito excreta

## Abstract

Arboviruses can be difficult to detect in the field due to relatively low prevalence in mosquito populations. The discovery that infected mosquitoes can release viruses in both their saliva and excreta gave rise to low-cost methods for the detection of arboviruses during entomological surveillance. We implemented both saliva and excreta-based entomological surveillance during the emergence of Zika virus (ZIKV) in French Guiana in 2016 by trapping mosquitoes around households of symptomatic cases with confirmed ZIKV infection. ZIKV was detected in mosquito excreta and not in mosquito saliva in 1 trap collection out of 85 (1.2%). One female *Ae. aegypti* L. (Diptera: Culicidae) was found with a ZIKV systemic infection in the corresponding trap. The lag time between symptom onset in a ZIKV-infected individual living near the trap site and ZIKV detection in this mosquito was 1 wk. These results highlight the potential of detection in excreta from trapped mosquitoes as a sensitive and cost-effective method to non invasively detect arbovirus circulation.

## Graphical Abstract

**Graphical Abstract:**
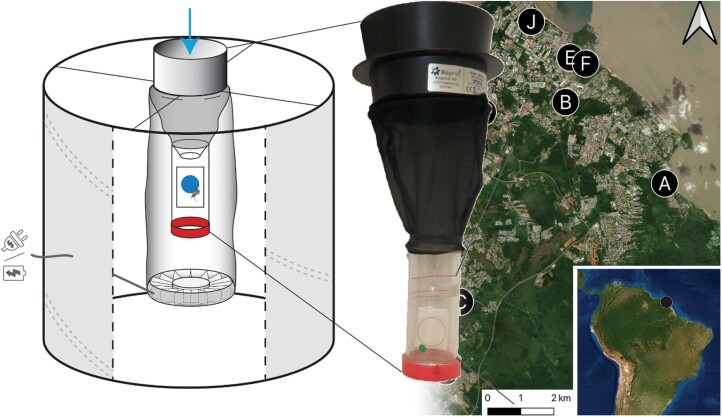

## Background

Growing global trade and travel activities are responsible for the global spread of arboviruses and their mosquito vectors, thereby increasing the threat of mosquito-borne diseases on human health worldwide (Wilder-Smith et al. 2017). The emergence of Zika virus (ZIKV) is the most recent example of how fast arboviruses can spread outside their endemic area to other regions where mosquito vectors are already present and environmental conditions are suitable (Fauci and Morens 2016). ZIKV was detected in South America in 2015 (Zanluca et al. 2015), with a first reported case in French Guiana in November 2015 (CIRE Antilles Guyane 2016). Although most ZIKV infections are asymptomatic or cause a mild self-limiting illness, the virus can be linked to neurological disorders (Brasil et al. 2016), severe congenital abnormalities, and human birth defects (Cauchemez et al. 2016, Rasmussen et al. 2016), leading the World Health Organization to declare a Public Health Emergency of International Concern (World Health Organization [WHO] 2016). ZIKV is transmitted to humans by mosquitoes, primarily *Aedes* (*Stegomyia* ) *aegypti* L. (Diptera: Culicidae) (Epelboin et al. 2017), but several recent studies have also highlighted that ZIKV can be transmitted between humans through sexual contact or from mother to fetus (Calvet et al. 2016, D’Ortenzio et al. 2016, de Laval et al. 2017).

The early detection of virus transmission can help in the implementation of adapted protective measures in areas where the risk of exposure is the highest. Current surveillance methods mainly involve case detection and seroprevalence studies in humans (Flamand et al. 2019) or detection of the virus in mosquito populations (i.e., entomological surveillance). An innovative entomological surveillance technique exploits the observation that infectious mosquitoes expectorate viruses in their saliva during sugar feeding (van den Hurk et al. 2007, 2014, Hall-Mendelin et al. 2010, Ibáñez-Justicia et al. 2012, Girod et al. 2016, Melanson et al. 2017, Kurucz et al. 2019, Wipf et al. 2019) and in their excreta (Fontaine et al. 2016, Ramírez et al. 2018, L’Ambert et al. 2023). The idea of surveillance based on these sample types is to attract mosquitoes to traps where they can feed and collectively expel virus particles in saliva or excreta onto paper filter cards which are treated with proprietary chemicals to preserve nucleic acids. Saliva and excreta-based methods have the benefit of making the processing of large quantities of trapped mosquitoes optional. Virus screening in mosquitoes can thus be undertaken only in traps where the virus has been detected in saliva or excreta, which greatly reduces cost and logistics compared with processing all captured mosquitoes.

Here, we implemented both saliva and excreta-based entomological surveillance during the emergence of ZIKV in French Guiana in 2016 by trapping mosquitoes around households of confirmed ZIKV symptomatic cases. Our results further extend the proof-of-concept of sugar feeding and excreta-based xenomonitoring to ZIKV.

## Materials and Methods

**Fig. 1:**
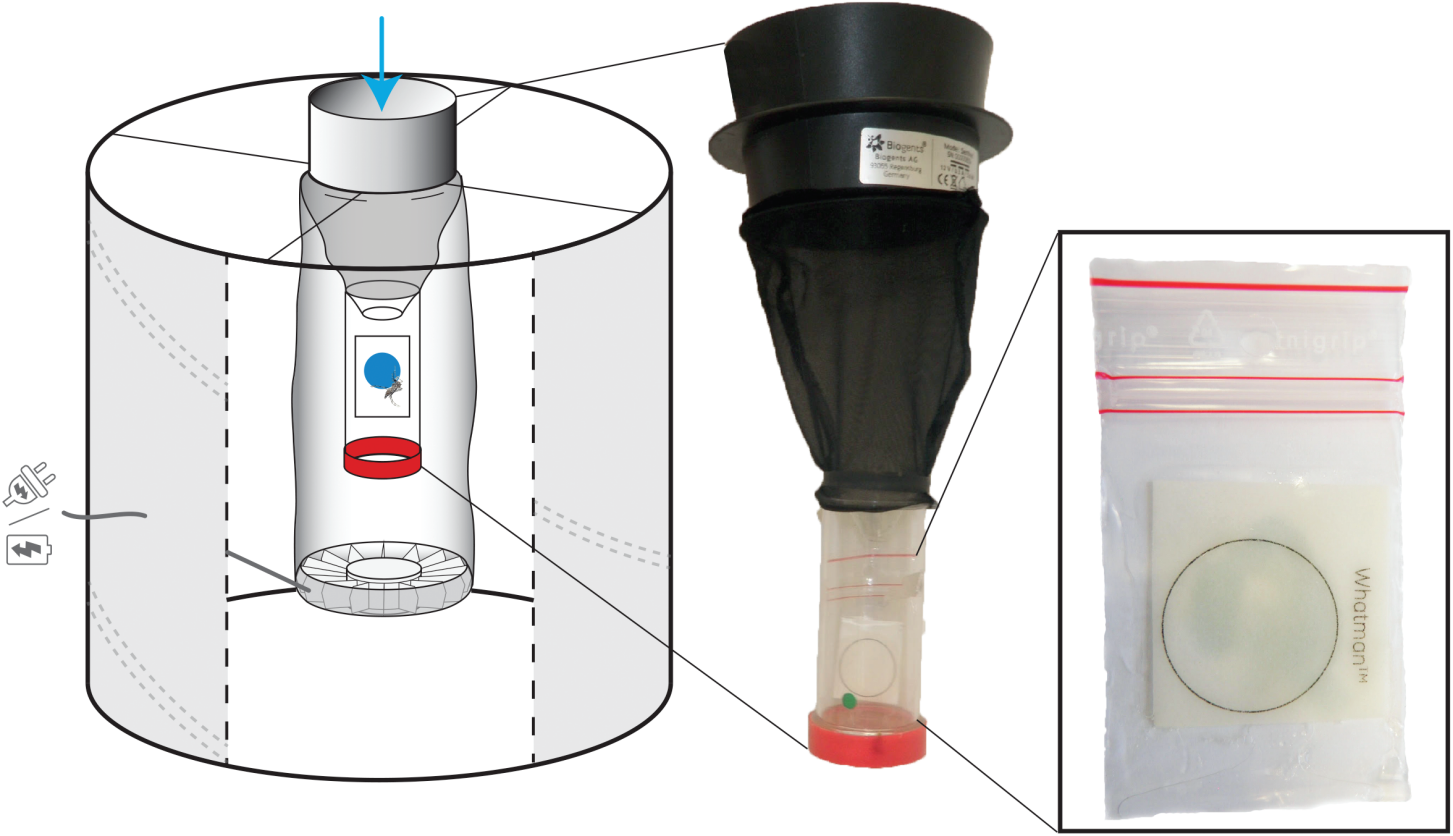
Schematic of the modified BG-Sentinel trap (Biogents). The trap was modified to incorporate a lodging for trapped mosquitoes with permanent access to colored honey impregnated in an FTA filter paper card to collect saliva, and a UFP placed at the bottom of the device to collect mosquito excreta.

BG-Sentinel (BG, Biogents AG, Regensburg, Germany) traps were modified to replace the original catch bag with lodging for trapped mosquitoes (Fig. 1). The lodging was made of a transparent 70 × 30 mm polypropylene screw cap tube (40 mL) (Dutscher, France, ref 688252) from which the bottom had been cut off. The tube was inverted with its open-end (cut section) directed upward and attached beneath the intake funnel. A colored honey-impregnated FTA (Flinders Technology Associates) card (Whatman, GE Healthcare, Florham Park, NJ, USA), placed in a small plastic bag with an opening at its center, was positioned on the tube wall to collect saliva during sugar feeding. An untreated filter paper (UFP) (Whatman, grade 3, ref. 1003-917) was placed at the bottom, inside the screwed cap, to collect mosquito excreta. Blue food coloring was added to honey to easily spot mosquito excreta on the UFP surface after sugar digestion. A BG-Lure (Biogents) attractant was placed in each trap.

**Fig. 2:**
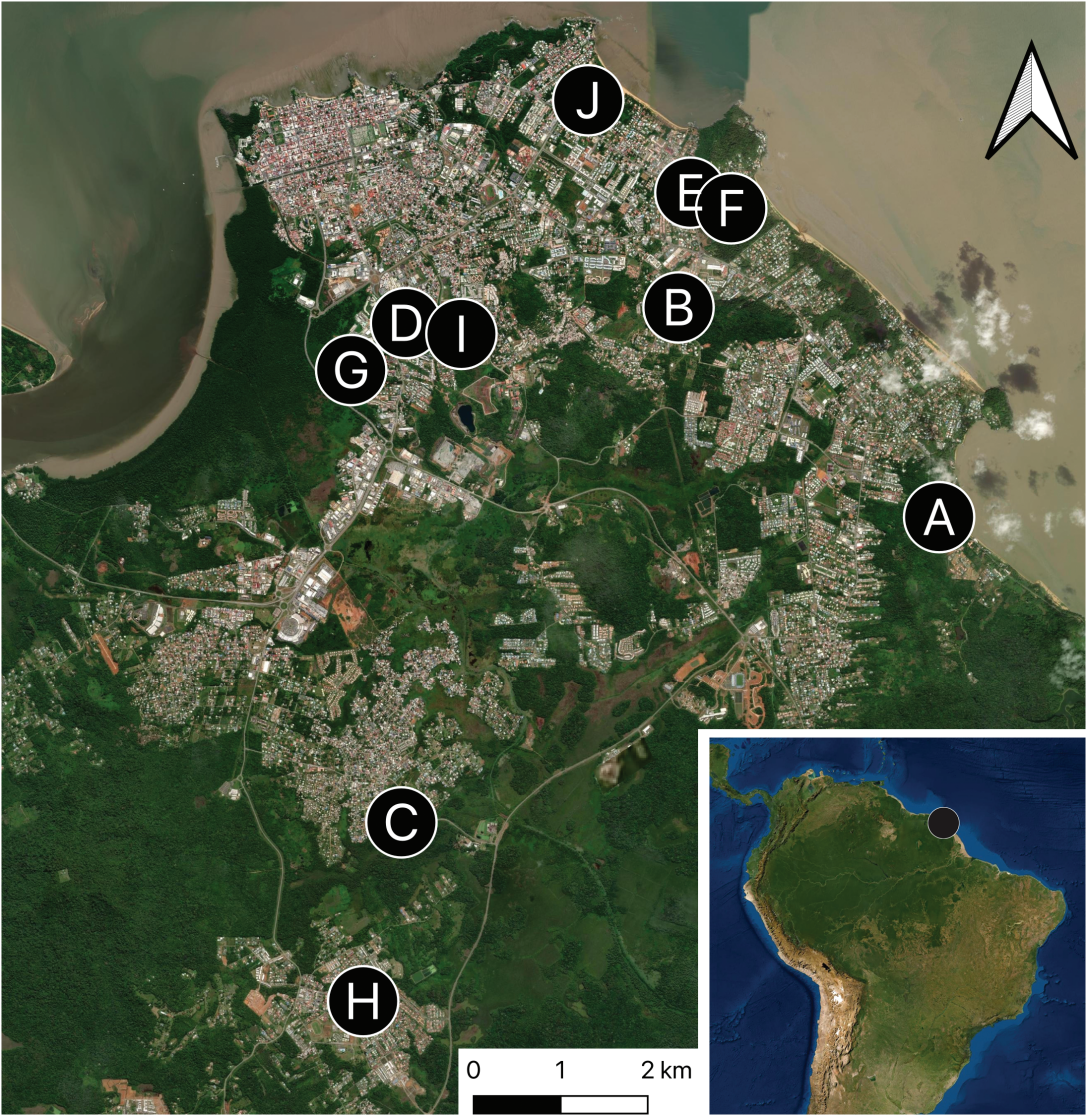
Study map. Modified BG-Sentinel traps (Biogents) localization in 10 distinct households of confirmed Zika virus cases on the French Guiana coastline. The map was created using the free and open source QGIS (Geographic Information System using satellite imagery) from the Environmental Systems Research Institute.

Modified BG traps were placed in 10 households of confirmed ZIKV-infected patients in Cayenne, Rémire-Montjoly, and Matoury cities (Fig. 2). The ZIKV-infected patients, who were already enrolled in a clinical study under the agreement number ID RCB: 2016-A00394-47 (de Laval et al. 2018), provided authorization for the placement of traps. A minimum of 2 traps per house, placed indoors and outdoors, were set during May and June 2016. After 3–7 consecutive days, the lodgings containing mosquitoes, FTA, and UFP cards were replaced. A total of 29 traps were monitored, and the procedure was repeated in each study site over a period of 4 wk. On the collection date, mosquitoes and cards from each site were transported to laboratory.

All mosquitoes were sorted by sex and identified at the species level based on the taxonomic keys of local species. The head and thorax of each mosquito were removed and placed individually into vials, while abdomens were pooled in 1.5 mL tubes to a maximum of 25 samples by species, site, and collection date. RNA extraction, reverse transcription, cDNA preamplification, and high-throughput real-time PCR were first performed following Moutailler et al. (2019) protocols on pools of mosquito abdomens. When ZIKV was detected in pools of abdomens, the cDNA of head/thorax of individual mosquitoes composing each pool was generated and screened for arboviruses by real-time PCR on a LightCycler 480 (LC480) (Roche Applied Science, Penzberg, Germany) (Moutailler et al. 2019). Sequencing was performed using the S5 Ion torrent technology (Thermo Fisher Scientific, Waltham, MA, USA) following manufacturer’s instructions.

FTA and UFP cards, exposed to trapped mosquitoes (i.e., potentially containing infected saliva), were cut and placed in 1.5 mL tubes containing 200 µL of DMEM (Dulbecco’s Modified Eagle Medium). After grinding with a sterile pestle, RNA was extracted from FTA and UFP homogenates using the QIAamp Viral RNA Mini Kit (Qiagen, Hilden, Germany) according to the manufacturer’s instructions. Detection of ZIKV genomic RNA was performed by amplifying a 77 bp genomic region coding for the envelope protein with a one-step reverse transcription real-time quantitative polymerase chain reaction (RT-qPCR) assay using the SuperScript III Platinum one-step RT-qPCR mix (Invitrogen, Cergy Pontoise, France). Reactions contained 0.5 µL of SuperScript III RT/Platinum Taq Mix, 12.5 µL of 2× Reaction Mix, 0.5 µL of ZIKV forward (5ʹ-CCGCTGCCCAACACAAG-3ʹ, primer 1086) and reverse (5ʹ-CCACTA ACGTTCTTTTGCAGACAT-3ʹ, primer 1162c) primers from Lanciotti et al. (2008) at 50 µM and 0.5 µL of a slightly modified probe (5ʹ-FAM-CCTACCTTGACAAGCARTCAGACAC-3ʹBHQ-1) at 10 µM, 5.5 µL of RNase-free water, and 5 μL of RNA in a final volume of 20 μL. Amplifications were performed on a StepOnePlus Real-Time PCR System (Applied Biosystems, Gaithersburg, MD, USA) using the following cycling protocol: 50 °C for 30 min, 95 °C for 2 min, 45 cycles at 95 °C for 15 s, 60 °C for 1 min.

## Results

A total of 1,379 mosquitoes were collected during the ZIKV epidemic, which affected French Guiana in 2016. Out of these mosquitoes, 521 (38%) were *Ae. aegypti.* Engorged *Ae. aegypti* females with a blue abdomen were detected in each site except for site A (Table 1). ZIKV was detected in 1 pool of abdomens out of 53 (2%) grouped by species, site, and collection date. This pool originated from mosquitoes trapped in Cayenne in site C on 9 June 2016. Heads and thoraxes from each mosquito corresponding to this pool were then individually screened for the presence of ZIKV. The virus was detected in the head and thorax of 1 female *Ae. aegypti* with a Cycle threshold (Ct) of 14. The full-length genome of this virus was sequenced (GenBank Accession number MN185326) and identified as the Asian genotype which is consistent with the strain circulating during the epidemic. This *Ae. aegypti* female was found dead in the trap and had a blue abdomen, indicative of a recent sugar meal. The time between symptom onset declared in the ZIKV-infected individual living in this site, and ZIKV detection in this mosquito was 1 wk. A total of 858 mosquitoes that were not *Ae. aegypti* species were also collected in this study. No ZIKV was detected in pooled abdomens (*N* = 80) from these mosquitoes (Supplementary Table S1).

**Table 1:** Summary of the entomological surveillance data of *Aedes aegypti* mosquitoes. Mosquito captures and collections of excreta and saliva cards were carried out in 10 houses from 12 May 2016 to 28 June 2016 spread across 3 cities where the Zika virus (ZIKV) was circulating. For each city, the total sample number (No.) of *Ae. aegypti* collections is indicated. The number of mosquitoes with blue abdomens (males and females combined) is also listed, indicating the ingestion of previously dyed blue honey on the filter paper card and, thus, the associated saliva deposition. The presence or absence of the ZIKV is also indicated in the abdomen pools of females, in saliva collection FTA filter paper cards, and in excreta collection UFP cards, with tested positive samples being indicated in bold in the table

Cities	No. of houses of mosquitoes collection (No. of traps)	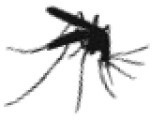				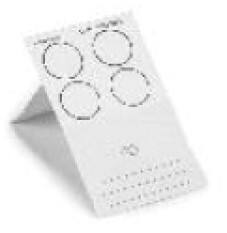	
		Field indication of mosquito salivation on cards		Screening viruses on females *Ae. Aegypti*		Screening ZIKV in cards	
		Total No. of *Ae. Aegypti*	Total No. of *Ae. Aegypti* with blue abdomen (percent density)	Total No. of females (percent density)	No. of abdomens pools with ZIKV/No. of pools of females tested	No. of cards with ZIKV in saliva/FTA cards processed	No. of cards with ZIKV in excreta/UFP cards processed
Cayenne	6 (18)	379	40 (11%)	183 (48%)	**1**/32	0/52	**1**/52
Rémire-Montjoly	2 (5)	60	5 (8%)	32 (53%)	0/7	0/13	0/12
Matoury	2 (6)	82	16 (20%)	54 (66%)	0/14	0/19	0/18
*Total*	*10 (29)*	*521*	*61 (12%)*	*269 (52%)*	*1/53*	*0/84*	*1/82*

A total of 166 cards out of the 226 cards collected during the study were processed after excluding cards from traps that did not contain mosquitoes. The virus was not detected in any of the 84 salivae FTA cards. ZIKV was detected in 1 excreta card out of the 82 excreta UFP cards with a Ct of 37. This card corresponded to the same trap in which the female *Ae. aegypti* infected with ZIKV was detected.

## Discussion

Although entomological surveillance aims to detect pathogens in the environment before symptomatic human cases emerge, its implementation faces challenges due to the need to process a substantial number of mosquitoes, the majority of which are uninfected, resulting in high costs and logistical constraints. Indeed, we observed a low virus detection rate even though traps were specifically placed in proximity to symptomatic human cases. Similar to previous studies (van den Hurk et al. 2007, 2014, Hall-Mendelin et al. 2010, Flies et al. 2015, Fontaine et al. 2016, Melanson et al. 2017, Ramírez et al. 2018, 2019, Meyer et al. 2019, L’Ambert et al. 2023), we have demonstrated that surveillance methods which exploit saliva or excreta can be used as an initial screen and whole mosquitoes processed only from traps which are positive.

Detecting viruses in the saliva is reflective of the presence of infectious vectors at a given place and date, thereby excluding any virus detection in digested blood or mosquitoes that are noninfectious or not yet infectious. In this study, the trapped female *Ae aegypti* infected with ZIKV had a blue abdomen, which was evidence of a recent sugar feeding on the colored FTA card. However, ZIKV was not detected on the FTA card but was detected in excreta. This could reflect the higher quantity of virus in excreta compared with that in saliva (Ramírez et al. 2018), or it was too early in the extrinsic incubation period for transmission to occur.

While detection of virus in excreta cannot be distinguished from virus present in species capable of transmission, it is indicative of the circulation of the pathogen in a specific location. In the future, DNA meta-barcoding strategies or high-throughput sequencing could be applied to excreta samples to provide information on the mosquito, vertebrate, and viral community diversity (Birnberg et al. 2020, Ramírez et al. 2020, L’Ambert et al. 2023). Overall, the detection of virus in trapped mosquito excreta is a promising tool in the development of a sustainable entomological surveillance system to prevent or limit the spread of arbovirus in a human population.

## Supplementary Material

Supplementary material is available at *Journal of Medical Entomology* online.

tjae016_suppl_Supplementary_Table_S1

## Data Availability

ZIKV genome is accessible under the *GenBank* accession number MN185326.
