# Supplementary material for: Noninvasive detection of Zika virus in mosquito excreta sampled from wild mosquito populations in French Guiana
Source: J Med Entomol. 2024 Feb 26;61(3):818–23. doi: 10.1093/jme/tjae016 (PMC11078575; doi:10.1093/jme/tjae016)
Supplement: tjae016_suppl_Supplementary_Table_S1 [file tjae016_suppl_supplementary_table_s1.docx]

**Supplementary Table S1: Information on the collection of mosquitoes other than *Aedes aegypti* by localization, date, and species. Numbers of total mosquitoes, of males/females, of alive/dead, and abdomen pools tested are shown.**

| **Cities** | **Collection date** | **Site** | **Genus** | **Species** | **Total number** | **No. of females** | **No. of males** | **No. of alives mosquitoes** | **No. of dead mosquitoes** | **No. of pools with ZIKV / No. of pools of females tested** |
| --- | --- | --- | --- | --- | --- | --- | --- | --- | --- | --- |
| **Remire-Montjoly** | 17-05-2016 | G | *Culex* | ***Culex sp.*** | **16** | 10 | 6 | 5 | 11 | 0/2 |
|  | 17-05-2016 | G | *Mansonia* | ***Mansonia titillans*** | **1** | 1 | 0 | 0 | 1 | 0/1 |
|  | 19-05-2016 | G | *Aedes* | ***Aedes scapularis*** | **1** | 1 | 0 | 0 | 1 | 0/1 |
|  | 19-05-2016 | G | *Culex* | ***Culex sp.*** | **10** | 4 | 6 | 4 | 6 | 0/1 |
|  | 23-05-2016 | G | *Aedes* | ***Aedes scapularis*** | **1** | 1 | 0 | 0 | 1 | 0/1 |
|  | 23-05-2016 | G | *Culex* | ***Culex sp.*** | **8** | 6 | 2 | 0 | 8 | 0/1 |
|  | 23-05-2016 | G | *Mansonia* | ***Mansonia titillans*** | **1** | 1 | 0 | 0 | 1 | 0/1 |
|  | 23-05-2016 | G | *Uranotaenia* | ***Uranotaenia sp.*** | **1** | 1 | 0 | 0 | 1 | 0/1 |
|  | 26-05-2016 | G | *Culex* | ***Culex sp.*** | **10** | 3 | 7 | 3 | 7 | 0/1 |
| **Matoury** | 23-05-2016 | I | *Coquillettidia* | ***Coquillettidia venezuelensis*** | **1** | 1 | 0 | 0 | 1 | 0/1 |
|  | 23-05-2016 | I | *Culex* | ***Culex sp.*** | **13** | 7 | 6 | 3 | 10 | 0/2 |
|  | 27-05-2016 | I | *Culex* | ***Culex sp.*** | **4** | 2 | 2 | 1 | 3 | 0/1 |
|  | 30-05-2016 | I | *Culex* | ***Culex sp.*** | **11** | 8 | 3 | 4 | 7 | 0/2 |
|  | 02-06-2016 | I | *Coquillettidia* | ***Coquillettidia venezuelensis*** | **1** | 1 | 0 | 0 | 1 | 0/1 |
|  | 02-06-2016 | I | *Culex* | ***Culex sp.*** | **2** | 1 | 1 | 1 | 1 | 0/1 |
|  | 13-06-2016 | J | *Culex* | ***Culex sp.*** | **1** | 1 | 0 | 0 | 1 | 0/1 |
|  | 16-06-2016 | J | *Culex* | ***Culex sp.*** | **2** | 1 | 1 | 0 | 2 | 0/1 |
|  | 20-06-2016 | J | *Culex* | ***Culex sp.*** | **2** | 2 | 0 | 0 | 2 | 0/1 |
|  | 23-06-2016 | J | *Coquillettidia* | ***Coquillettidia venezuelensis*** | **2** | 2 | 0 | 0 | 2 | 0/1 |
|  | 23-06-2016 | J | *Culex* | ***Culex sp.*** | **1** | 1 | 0 | 0 | 1 | 0/1 |
| **Cayenne** | 24-05-2016 | A | *Culex* | ***Culex sp.*** | **18** | 4 | 14 | 1 | 17 | 0/1 |
|  | 26-05-2016 | A | *Culex* | ***Culex sp.*** | **5** | 1 | 4 | 3 | 2 | 0/1 |
|  | 31-05-2016 | A | *Coquillettidia* | ***Coquillettidia venezuelensis*** | **1** | 1 | 0 | 0 | 1 | 0/1 |
|  | 31-05-2016 | A | *Culex* | ***Culex sp.*** | **90** | 46 | 44 | 32 | 58 | 0/2 |
|  | 02-06-2016 | A | *Culex* | ***Culex sp.*** | **48** | 21 | 27 | 11 | 37 | 0/1 |
| **Cities** | **Collection date** | **Site** | **Genus** | **Species** | **Total number** | **No. of females** | **No. of males** | **No. of alives mosquitoes** | **No. of dead mosquitoes** | **No. of pools with ZIKV / No. of pools of females tested** |
| **Cayenne** | 09-06-2016 | B | *Culex* | ***Culex sp.*** | **89** | 46 | 43 | 14 | 75 | 0/3 |
|  | 09-06-2016 | C | *Coquillettidia* | ***Coquillettidia venezuelensis*** | **2** | 2 | 0 | 0 | 2 | 0/1 |
|  | 09-06-2016 | C | *Culex* | ***Culex sp.*** | **8** | 3 | 5 | 0 | 8 | 0/2 |
|  | 09-06-2016 | D | *Coquillettidia* | ***Coquillettidia venezuelensis*** | **7** | 7 | 0 | 1 | 6 | 0/1 |
|  | 09-06-2016 | D | *Culex* | ***Culex sp.*** | **6** | 4 | 2 | 4 | 2 | 0/2 |
|  | 09-06-2016 | D | *Mansonia* | ***Mansonia titillans*** | **3** | 3 | 0 | 1 | 2 | 0/1 |
|  | 13-06-2016 | B | *Aedes* | ***Aedes scapularis*** | **1** | 1 | 0 | 0 | 1 | 0/1 |
|  | 13-06-2016 | B | *Culex* | ***Culex sp.*** | **106** | 62 | 44 | 40 | 66 | 0/5 |
|  | 13-06-2016 | C | *Culex* | ***Culex sp.*** | **8** | 2 | 6 | 1 | 7 | 0/2 |
|  | 14-06-2016 | D | *Coquillettidia* | ***Coquillettidia venezuelensis*** | **3** | 3 | 0 | 0 | 3 | 0/2 |
|  | 14-06-2016 | D | *Culex* | ***Culex sp.*** | **59** | 18 | 41 | 14 | 45 | 0/3 |
|  | 14-06-2016 | D | *Uranotaenia* | ***Uranotaenia apicalis*** | **2** | 2 | 0 | 0 | 2 | 0/1 |
|  | 16-06-2016 | B | *Aedes* | ***Aedes taeniorhynchus*** | **1** | 1 | 0 | 0 | 1 | 0/1 |
|  | 16-06-2016 | B | *Coquillettidia* | ***Coquillettidia venezuelensis*** | **1** | 1 | 0 | 0 | 1 | 0/1 |
|  | 16-06-2016 | B | *Culex* | ***Culex sp.*** | **53** | 18 | 35 | 19 | 34 | 0/1 |
|  | 16-06-2016 | C | *Culex* | ***Culex sp.*** | **6** | 4 | 2 | 2 | 4 | 0/1 |
|  | 16-06-2016 | D | *Coquillettidia* | ***Coquillettidia venezuelensis*** | **1** | 1 | 0 | 0 | 1 | 0/1 |
|  | 16-06-2016 | D | *Culex* | ***Culex sp.*** | **19** | 10 | 9 | 12 | 7 | 0/5 |
|  | 16-06-2016 | F | *Culex* | ***Culex sp.*** | **15** | 4 | 11 | 4 | 11 | 0/1 |
|  | 20-06-2016 | B | *Coquillettidia* | ***Coquillettidia venezuelensis*** | **2** | 1 | 1 | 0 | 2 | 0/1 |
|  | 20-06-2016 | B | *Culex* | ***Culex sp.*** | **95** | 40 | 55 | 21 | 74 | 0/3 |
|  | 20-06-2016 | B | *Limatus* | ***Limatus durhami*** | **1** | 1 | 0 | 0 | 1 | 0/1 |
|  | 20-06-2016 | E | *Culex* | ***Culex sp.*** | **2** | 2 | 0 | 0 | 2 | 0/1 |
|  | 20-06-2016 | F | *Culex* | ***Culex sp.*** | **27** | 5 | 22 | 5 | 22 | 0/1 |
|  | 20-06-2016 | F | *Limatus* | ***Limatus durhami*** | **2** | 2 | 0 | 0 | 2 | 0/1 |
| **Cities** | **Collection date** | **Site** | **Genus** | **Species** | **Total number** | **No. of females** | **No. of males** | **No. of alives mosquitoes** | **No. of dead mosquitoes** | **No. of pools with ZIKV / No. of pools of females tested** |
| **Cayenne** | 21-06-2016 | D | *Anopheles* | ***Anopheles aquasalis*** | **1** | 1 | 0 | 0 | 1 | 0/1 |
|  | 21-06-2016 | D | *Coquillettidia* | ***Coquillettidia venezuelensis*** | **13** | 13 | 0 | 0 | 13 | 0/1 |
|  | 21-06-2016 | D | *Culex* | ***Culex portesi*** | **3** | 3 | 0 | 0 | 3 | 0/1 |
|  | 21-06-2016 | D | *Culex* | ***Culex sp.*** | **54** | 16 | 38 | 12 | 42 | 0/3 |
|  | 21-06-2016 | D | *Mansonia* | ***Mansonia titillans*** | **2** | 2 | 0 | 1 | 1 | 0/1 |
|  | 23-06-2016 | E | *Culex* | ***Culex sp.*** | **15** | 1 | 14 | 7 | 8 | 0/1 |
|  |  |  |  | ***Total*** | ***858*** | ***407*** | ***451*** | ***226*** | ***632*** | 0/80 |
